# Identification and validation of aging related genes in osteoarthritis

**DOI:** 10.3389/fgene.2025.1561644

**Published:** 2025-05-30

**Authors:** Jian Du, Tian Zhou, Yanghui Dong, Yunchao Sun, Wei Peng

**Affiliations:** ^1^ Senior Department of Orthopedics, The Fourth Medical Center of PLA General Hospital, Beijing, China; ^2^ Hebei North University, Zhangjiakou, China

**Keywords:** osteoarthritis, aging related genes (ARGs), machine learning, key genes, prediction model, immune cell infiltration

## Abstract

**Background:**

Osteoarthritis (OA) is a degenerative disease associated with aging. Although an increasing body of research suggests a close relationship between aging and OA, the underlying mechanisms remain unclear. This study explores the relationship between aging related genes (ARGs) and OA, providing potential new targets for understanding the pathogenesis and treatment of OA.

**Methods:**

The OA synovial tissue dataset was obtained from the GEO database, and differentially expressed genes (DEGs) were screened. The DEGs were intersected with ARGs to identify differentially expressed aging related genes (DEARGs), which were then subjected to functional enrichment analysis, PPI network analysis, and machine learning algorithms (LASSO and RF) to identify key genes. In addition, a nomogram was constructed based on the key genes to predict OA risk, and its diagnostic value was evaluated using ROC curves. Subsequently, the expression levels of the key genes were validated through qRT-PCR experiments. Finally, the CIBERSORT algorithm was applied to assess the proportion of immune cells and investigate the correlation between the key genes and immune cells.

**Results:**

A total of 34 DEARGs were identified. PPI network analysis revealed 12 key DEARGs. Subsequently, LASSO and RF algorithms identified ATF3, KLF4, NFKBIA, and SOD2 as key genes. Based on nomogram and ROC curve analysis, these four key genes demonstrated good diagnostic value. qRT-PCR showed that ATF3, KLF4, NFKBIA, and SOD2 were significantly downregulated in OA. Immune infiltration analysis revealed differences in Plasma cells, T cells follicular helper, Mast cells resting, T cells CD4 memory resting, NK cells activated, Monocytes, and Mast cells activated between the OA group and normal controls.

**Conclusion:**

ATF3, KLF4, NFKBIA and SOD2 are identified as novel biomarkers associated with aging in OA and may serve as potential therapeutic targets for OA treatment.

## Introduction

Osteoarthritis (OA) is a common degenerative joint disease in the elderly, characterized primarily by synovial inflammation and cartilage degradation, often resulting in joint pain, stiffness, and functional limitations (Sellam and Berenbaum, 2010). With the continuous increase in the aging global population, the prevalence of OA is also steadily rising (Wei et al., 2023; Giorgino et al., 2023). It is estimated that approximately 250 million people worldwide suffer from OA (Hunter and Bierma-Zeinstra, 2019). Currently, treatment strategies for OA mainly include pharmacological therapy, physical therapy, and surgical intervention (Wang et al., 2025). However, these treatments only alleviate symptoms and do not provide a complete cure (Abramoff and Caldera, 2020). Therefore, a deeper understanding of the pathogenesis of OA is essential for developing more effective intervention and prevention strategies.

Although OA has long been considered a degenerative disease primarily caused by cartilage wear, it is now recognized as a complex pathological process (Man and Mologhianu, 2014). Increasing evidence suggests that synovitis plays a critical role in the pathogenesis of OA (Zhao et al., 2023). The synovial tissue is composed of an intimal lining layer and a sublining layer, and it plays a vital role in maintaining joint homeostasis (Han et al., 2020). The intimal lining layer contains synovial macrophages and synovial fibroblasts (Wu et al., 2024). In OA synovium, synovial macrophages exacerbate local inflammation and promote cartilage matrix degradation by secreting pro-inflammatory cytokines (Xu and Ji, 2023). Meanwhile, synovial fibroblasts contribute to extracellular matrix breakdown by releasing matrix metalloproteinases, thereby participating in joint tissue remodeling (Pap et al., 2020). These findings highlight the important role of synovial tissue in the pathological progression of OA.

Aging is a progressive decline in physiological function at both the tissue and cellular levels (Han et al., 2024). One of the core features of aging is cellular senescence, which is characterized by irreversible cell cycle arrest and the secretion of pro-inflammatory factors into the surrounding microenvironment. This phenomenon is known as the senescence-associated secretory phenotype (SASP) (Childs et al., 2015; Muñoz-Espín and Serrano, 2014). The persistent presence of SASP can induce chronic low-grade inflammation, further promoting senescence in neighboring cells and accelerating the overall aging process (Rodier et al., 2009). An increasing number of studies have indicated that aging plays a critical role in the development of OA (Ansari et al., 2024). However, the precise molecular relationship between aging and OA remains unclear. Therefore, further elucidating the role of aging in OA pathogenesis is of significant scientific importance.

**FIGURE 1:**
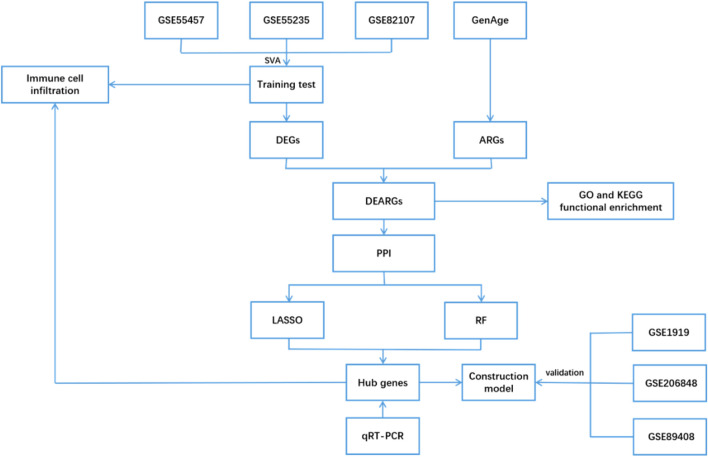
The flow chart of the analyses.

In this study, we screened aging-related differentially expressed genes (DEGs) associated with OA by integrating data from the GEO and CellAge databases. Protein–protein interaction (PPI) networks and machine learning algorithms were then used to identify key genes, providing a theoretical foundation for further understanding the pathogenesis of OA and exploring potential therapeutic strategies. The detailed workflow of this study is illustrated in Figure 1.

## Materials and methods

### Data collection and processing

Data were retrieved from the GEO database (http://www.ncbi.nlm.nih.gov/geo/) using the keywords “osteoarthritis,” “*Homo sapiens*,” and “expression profiles by array.” Datasets of normal synovial tissue and OA synovial tissue, including GSE55235, GSE55457, GSE82107, GSE1919, GSE206848, and GSE89408, were obtained. The detailed information of the dataset can be found in Table 1. Probe annotation files were used to convert probes into gene symbols. R software’s “limma” package was then applied for background correction and normalization of each dataset. Additionally, the “sva” package was used to merge the gene expression data of the GSE55235, GSE55457, and GSE82107 datasets into a single gene expression profile (training set) to remove batch effects for subsequent analysis (Leek et al., 2012). Meanwhile, GSE1919, GSE206848, and GSE89408 were used as the validation sets.

**TABLE 1:** The details of gene expression datasets.

Dataset	Platform	Samples	OA	Normal	Class
GSE55235	GPL96	Synovium	10	10	Train
GSE55457	GPL96	Synovium	10	10	Train
GSE82107	GPL570	Synovium	10	7	Train
GSE1919	GPL91	Synovium	5	5	Test
GSE206848	GPL570	Synovium	7	7	Test
GSE89408	GPL11154	Synovium	22	28	Test

### Identification of ARDEGs

Differential expression analysis was performed using the “limma” package in R software, with a threshold of adjusted p-value < 0.05 and |log2FC| > 1. DEGs were visualized using the “ggplot2” and “heatmap” packages (Ritchie et al., 2015). Aging-related genes were obtained from the Human Ageing Genomic Resources (GenAge, https://genomics.senescence.info/). Overlapping genes between DEGs and aging-related genes were identified as ARDEGs, and a Venn diagram was generated using the “VennDiagram” package.

### Functional enrichment analysis

Gene Ontology (GO) and Kyoto Encyclopedia of Genes and Genomes (KEGG) enrichment analyses of ARDEGs were performed using the “clusterProfiler” package in R software (Chen et al., 2017). The GO enrichment analysis was divided into three categories: biological process (BP), cellular component (CC), and molecular function (MF). Additionally, the results were visualized using the “ggplot2” package in R software, and a p-value <0.05 was considered statistically significant.

### PPI network construction and analysis

PPI analysis of ARDEGs was performed using the STRING database (https://cn.string-db.org/), with a minimum interaction score set to 0.4 (Szklarczyk et al., 2023). The PPI network was then constructed using Cytoscape (version 3.9.1) software, and the MCODE plugin within Cytoscape was employed to identify key ARDEGs for subsequent analysis.

### Machine learning for key genes selection

To further identify key genes from the ARDEGs, we employed two machine learning algorithms: Least Absolute Shrinkage and Selection Operator (LASSO) and Random Forest (RF). The LASSO algorithm minimizes the absolute values of regression coefficients and removes redundant or irrelevant genes, effectively reducing the risk of overfitting (Friedman et al., 2010). The LASSO algorithm was performed using the “glmnet” package in R software, with the optimal λ parameter determined by 10-fold cross-validation. The minimum λ value was selected to determine the number of genes to be filtered. RF is a robust predictive algorithm that improves prediction accuracy by aggregating multiple decision trees (Gao et al., 2024). The RF algorithm was executed using the “randomForest” package in R, with genes having an importance score greater than 2 considered the most valuable. Finally, the intersection of results from both machine learning methods was used to identify key genes.

### Construction and evaluation of the key genes prediction model

To enhance clinical applicability, we constructed a key gene nomogram prediction model using the “rms” package in R software (Zhang et al., 2024). In this model, “Points” represent the individual score for each candidate gene, while “Total Points” is the sum of the scores for all genes. Subsequently, we evaluated the accuracy of the model through Decision Curve Analysis (DCA), calibration curves, and ROC curves, including the Area Under the Curve (AUC) values. Additionally, the robustness and reliability of the model were further validated using three external datasets (GSE1919, GSE206848, and GSE89408).

### Evaluation of immune cell infiltration

Immune cell infiltration in both the normal control group and the OA group was assessed using the CIBERSORT algorithm, which estimates the relative proportions of 22 distinct immune cell types (Song et al., 2019). To further analyze the differences in immune cell between the two groups, a box plot was generated using the “ggplot2” package in R software. Additionally, the correlations among the 22 immune cell types were visualized in a heatmap created with the “corrplot” package in R software.

### Correlation analysis between key genes and immune cells

To further explore the relationship between key genes and immune cells, we employed the “ggplot2” package in R software to create a lollipop plot illustrating the correlations between the key genes and immune cell types.

### Clinical sample collection

In this study, synovial tissue samples were collected from 5 patients with meniscal injury (Control group) and 6 patients undergoing total knee arthroplasty for OA (OA group). The inclusion criteria were as follows: (1) diagnosis of OA according to the criteria established by the Chinese Orthopedic Association; (2) patient age between 45 and 65 years; (3) provision of written informed consent. The exclusion criteria included: (1) patients with autoimmune diseases such as rheumatoid arthritis or systemic lupus erythematosus; (2) patients with serious infections, hypertension, coronary artery disease, diabetes, or other significant comorbidities. Furthermore, this study was approved by the Ethics Committee of the Eighth Medical Center of the PLA General Hospital and conducted in accordance with the principles of the Declaration of Helsinki.

### qRT-PCR experimental validation

Total RNA was extracted from the synovial tissues using Trizol reagent (Servicebio), and reverse transcription was performed with Takara Prime Script^®^ RT Master Mix to synthesize cDNA. qRT-PCR was then carried out using 2 × SYBR Green qPCR Mix, and the relative expression levels of the genes were calculated using the 2^−ΔΔCT^ method. The primer sequences used for the qRT-PCR assays are listed in Table 2.

**TABLE 2:** Primer Sequences for qRT-PCR.

Gene	Forward primer (5′–3′)	Reverse primer (5′–3′)
GAPDH	CAT​GTA​CGT​TGC​TAT​CCA​GGC	CTC​CTT​AAT​GTC​ACG​CAC​GAT
ATF3	CCT​CTG​CGC​TGG​AAT​CAG​TC	TTC​TTT​CTC​GTC​GCC​TCT​TTT​T
KLF4	CGG​ACA​TCA​ACG​ACG​TGA​G	GAC​GCC​TTC​AGC​ACG​AAC​T
NFKBIA	CTC​CGA​GAC​TTT​CGA​GGA​AAT​AC	GCC​ATT​GTA​GTT​GGT​AGC​CTT​CA
SOD2	GCT​CCG​GTT​TTG​GGG​TAT​CTG	GCG​TTG​ATG​TGA​GGT​TCC​AG

### Statistical analysis

Experimental data were processed and analyzed using R software (version 4.2.1) and GraphPad Prism 9. Correlation analysis was performed using Pearson’s correlation test. Differences between the two groups were compared using an independent sample t-test, with p < 0.05 considered statistically significant.

## Results

### Identification of ARDEGs

**FIGURE 2:**
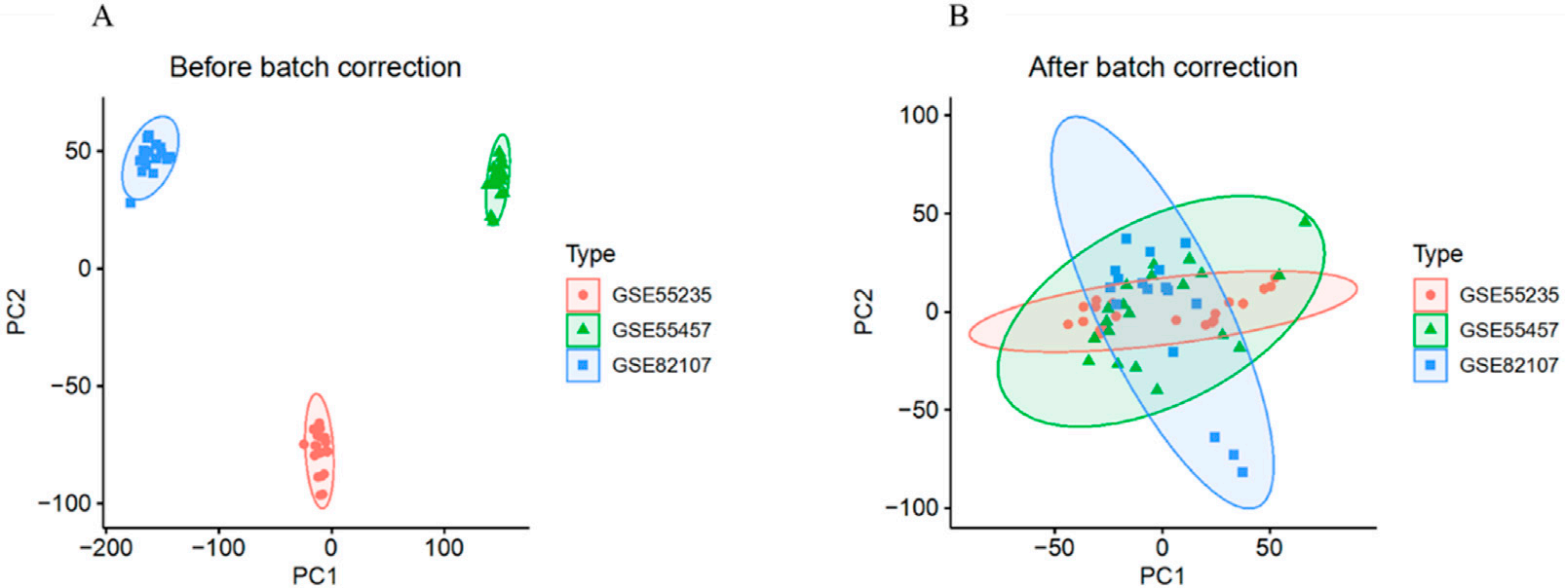
Removal of Batch Effects. **(A)** Distribution of the three datasets before batch effect removal. **(B)** Distribution of the three datasets after batch effect removal.

**FIGURE 3:**
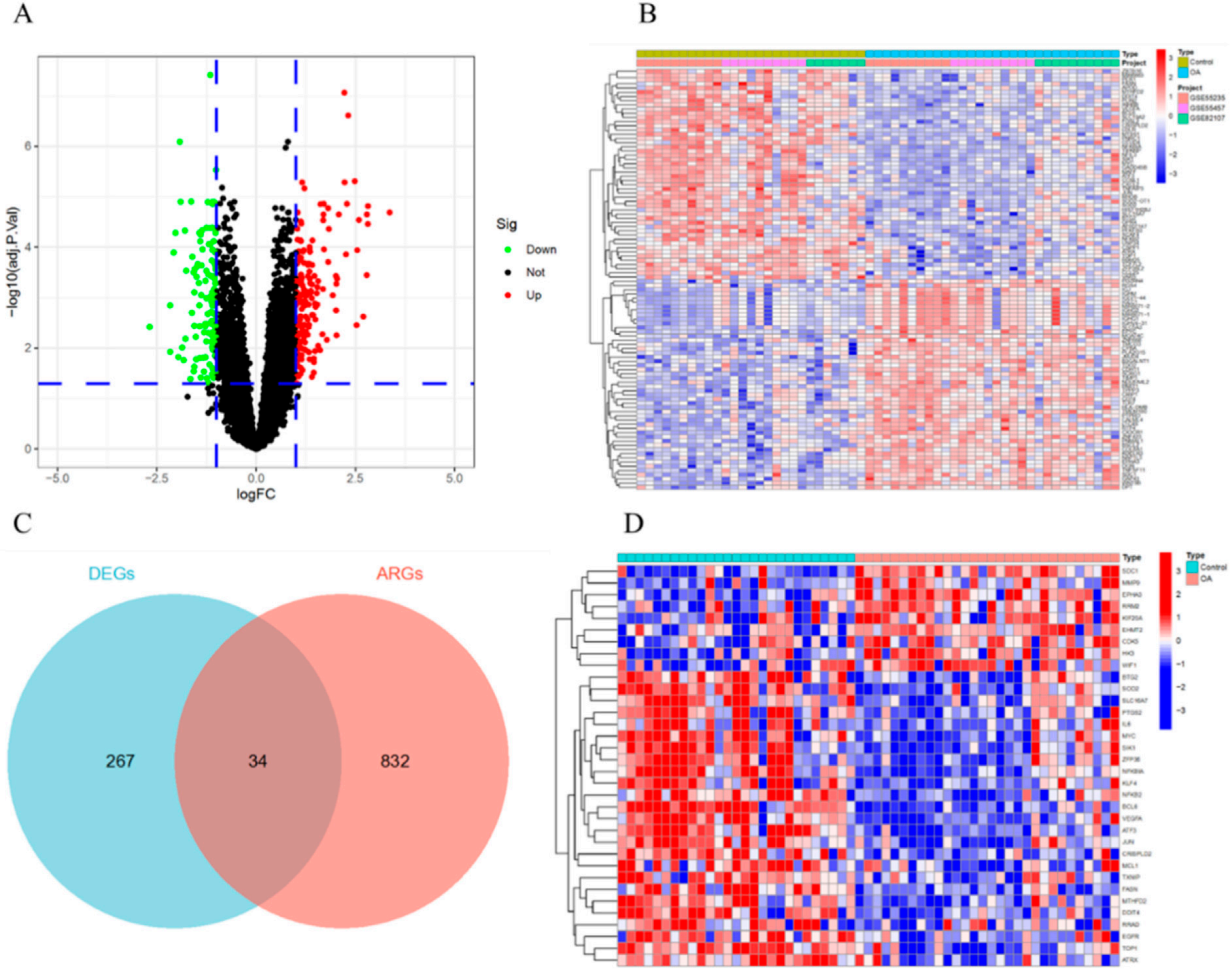
Identification of ARDEGs. **(A)** Volcano plot of DEGs. **(B)** Heatmap of the top 50 DEGs. **(C)** Intersection of DEGs and ARGs. **(D)** Heatmap of the 34 ARDEGs.

After removing batch effects, we merged the gene expression profiles from the GSE55235, GSE55457, and GSE82107 datasets into a single gene expression data set (Figures 2A,B). Differential expression analysis identified a total of 301 DEGs, including 178 upregulated and 123 downregulated genes (Figures 3A,B). Furthermore, by intersecting the DEGs with aging ARGs, we identified 34 ARDEGs (Figure 3C), which comprised 9 upregulated genes and 25 downregulated genes (Figure 3D).

### Functional enrichment analysis of ARDEGs

**FIGURE 4:**
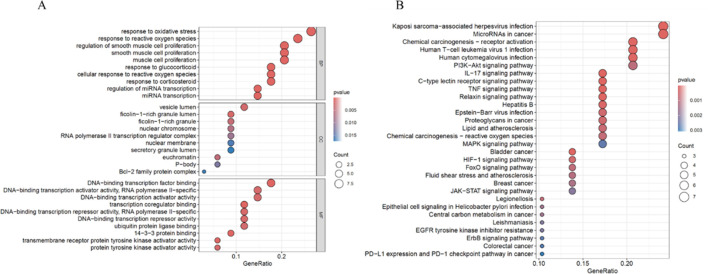
Functional Enrichment Analysis of ARDEGs. **(A)** Bubble plot of GO enrichment analysis for ARDEGs in BP, CC, and MF. **(B)** Bubble plot of KEGG pathway enrichment analysis for ARDEGs.

GO and KEGG pathway enrichment analyses were performed on ARDEGs to explore their potential biological functions. The results of the GO enrichment analysis showed that in BP, ARDEGs were enriched in processes such as response to oxidative stress, response to reactive oxygen species, and regulation of smooth muscle cell proliferation. In CC, ARDEGs were enriched in processes related to vesicle lumen, ficolin-1-rich granule lumen, and ficolin-1-rich granule. In MF, ARDEGs were enriched in processes such as DNA-binding transcription factor binding, DNA-binding transcription activator activity, RNA polymerase II-specific, and DNA-binding transcription activator activity (Figure 4A).

The KEGG enrichment analysis revealed that DEARGs were mainly enriched in pathways such as Kaposi sarcoma-associated herpesvirus infection, MicroRNAs in cancer, Human T-cell leukemia virus 1 infection, PI3K-Akt signaling pathway, and IL-17 signaling pathway (Figure 4B).

### PPI network construction and analysis

**FIGURE 5:**
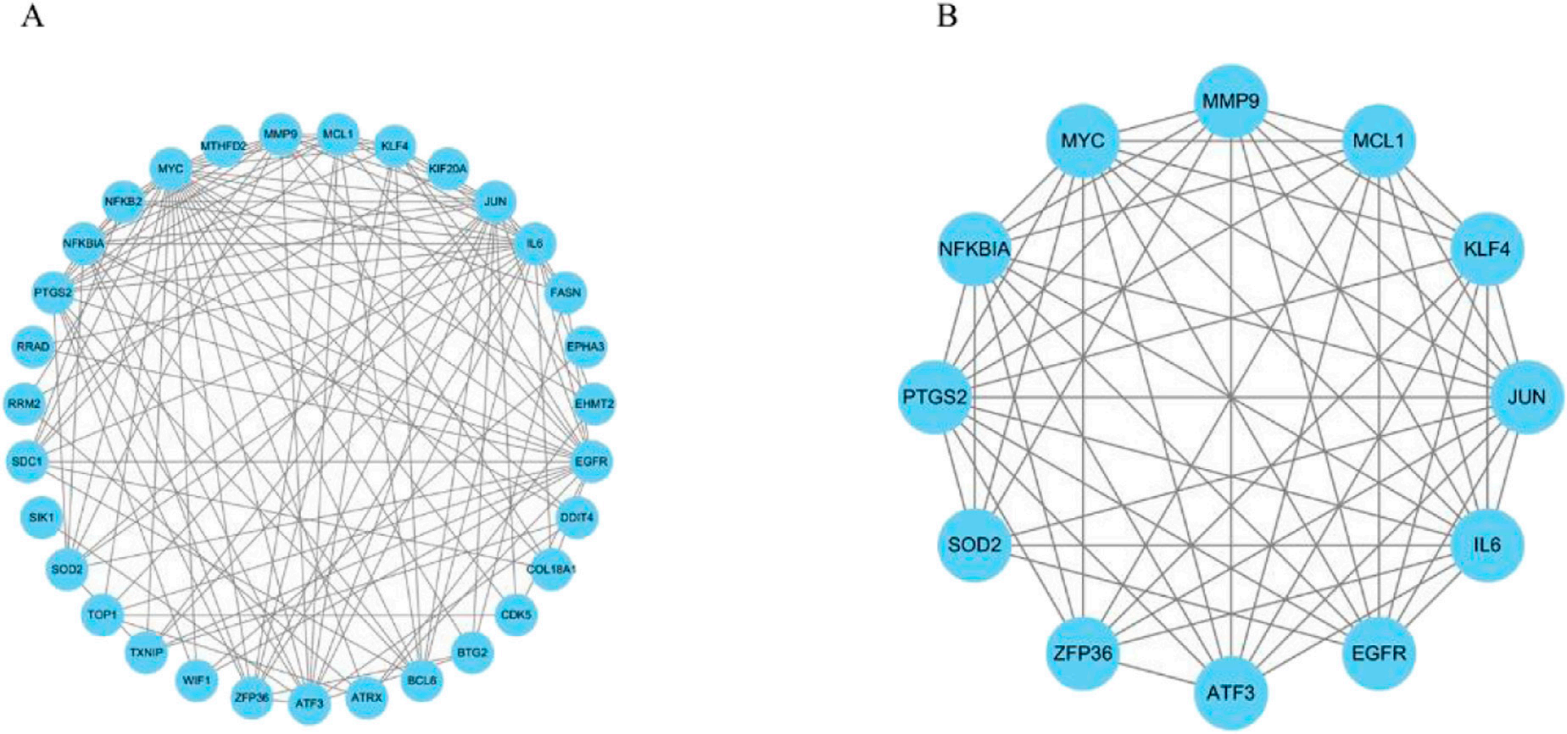
PPI Network Construction and Analysis. **(A)** PPI network of ARDEGs. **(B)** A cluster containing 12 key ARDEGs identified by MCODE.

To explore the interactions between ARDEGs, PPI network was constructed using Cytoscape software, which included 31 nodes and 126 edges (Figure 5A). Based on the MCODE plugin in Cytoscape, a cluster was identified (Figure 5B), consisting of 12 key ARDEGs: MMP9, MCL1, KLF4, JUN, IL6, EGFR, ATF3, ZFP36, SOD2, PTGS2, NFKBIA and MYC.

### Machine learning screening for key genes

**FIGURE 6:**
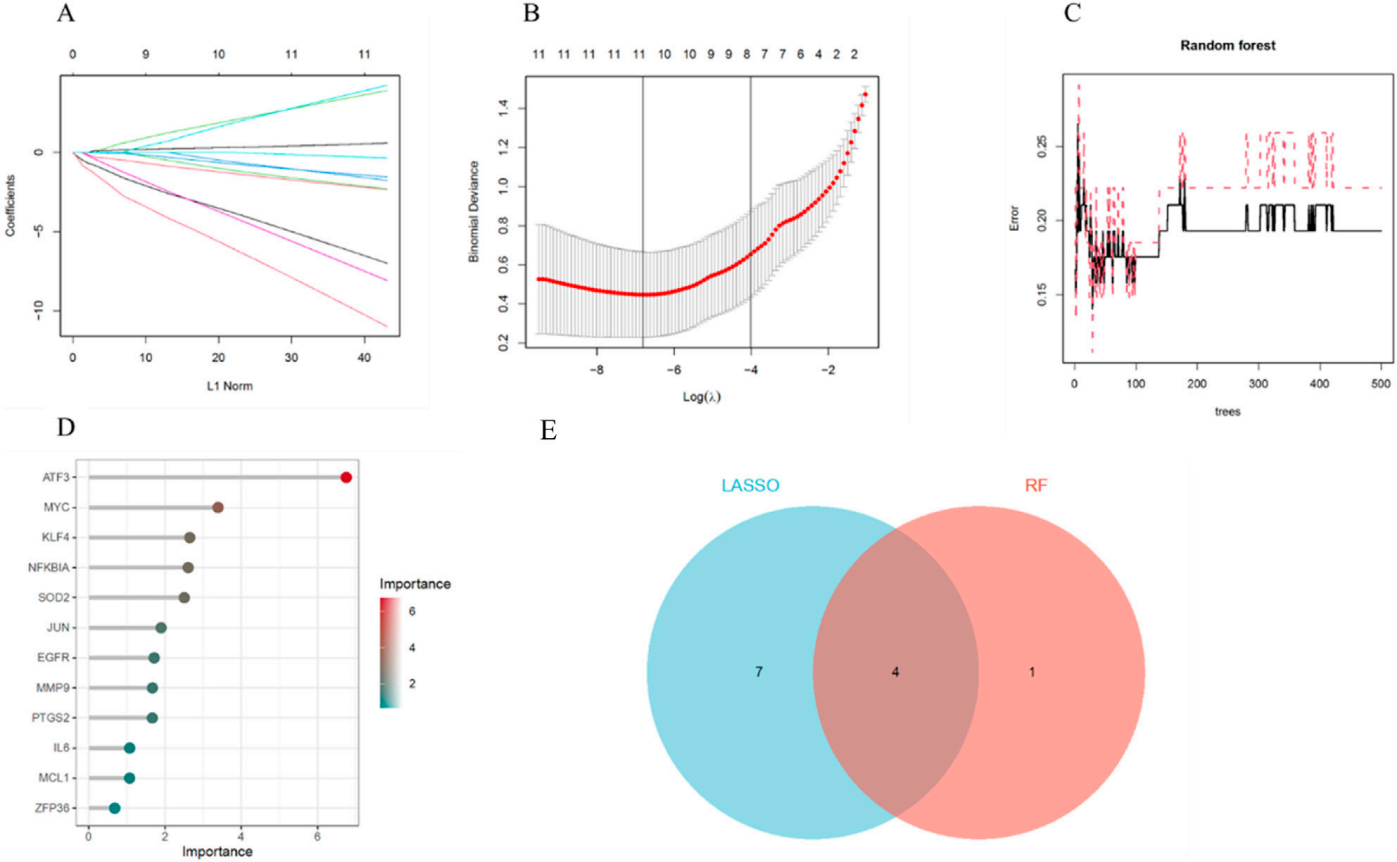
Machine Learning Screening for Key Genes. **(A)** LASSO coefficient analysis. **(B)** 10-fold cross-validation for selecting the optimal parameter in the LASSO model. **(C)** Relationship between the number of trees and error rate in the RF model. **(D)** Ranking of importance scores in the RF model. **(E)** Identification of key genes through the intersection of results from the two machine learning algorithms.

To enhance the reliability of the results, we employed two machine learning algorithms to further identify key aging-related genes. The LASSO algorithm selected 11 aging-related genes from the 12 key ARDEGs, including ATF3, EGFR, IL6, JUN, KLF4, MCL1, MMP9, NFKBIA, PTGS2, SOD2, and ZFP36 (Figures 6A,B). Based on importance scores greater than 2, the RF algorithm identified 5 aging-related genes: ATF3, MYC, KLF4, NFKBIA and SOD2 (Figures 6C,D). Ultimately, by intersecting the results of LASSO and RF, we identified 4 key genes (ATF3, KLF4, NFKBIA and SOD2) (Figure 6E).

### Construction of the key gene prediction model

**FIGURE 7:**
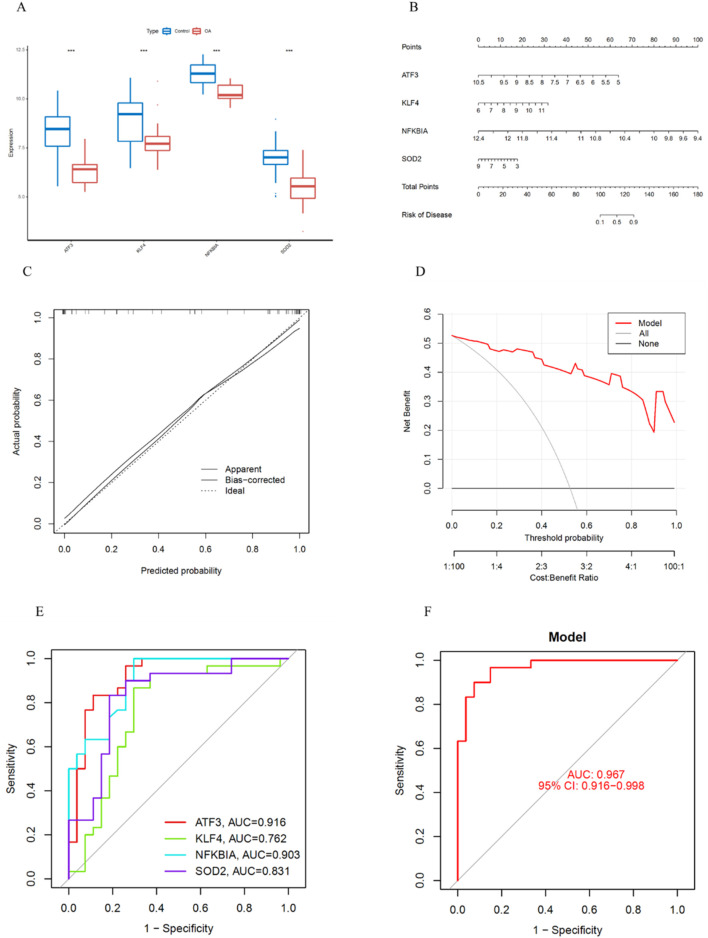
Construction of the Nomogram Prediction Model and ROC Curve Analysis. **(A)** Expression levels of key genes in the training set. **(B)** Construction of the nomogram prediction model. **(C)** Calibration curve of the nomogram prediction model. **(D)** DCA of the nomogram prediction model. **(E)** ROC curves of the four key genes in the training set. **(F)** ROC curve of the prediction model.

First, we validated the expression levels of the four key genes in the training set. The results showed that ATF3, KLF4, NFKBIA and SOD2 were significantly downregulated in OA (Figure 7A). Next, we constructed a risk prediction model using these four key genes. The nomogram score was used to predict the risk of OA development (Figure 7B). Additionally, the calibration curve demonstrated the accuracy of the model in predicting OA (Figure 7C). DCA indicated that the model had high predictive ability in OA diagnosis (Figure 7D). We also performed ROC curve analysis to assess the diagnostic performance of each gene in the risk prediction model. The training set results showed that the AUC values for ATF3, KLF4, NFKBIA and SOD2 were 0.916, 0.762, 0.903 and 0.813, respectively (Figure 7E). Furthermore, the nomogram showed that the model had high diagnostic performance, with an AUC of 0.967 (95% CI: 0.916–0.988) (Figure 7F).

### Validation of the prediction model

**FIGURE 8:**
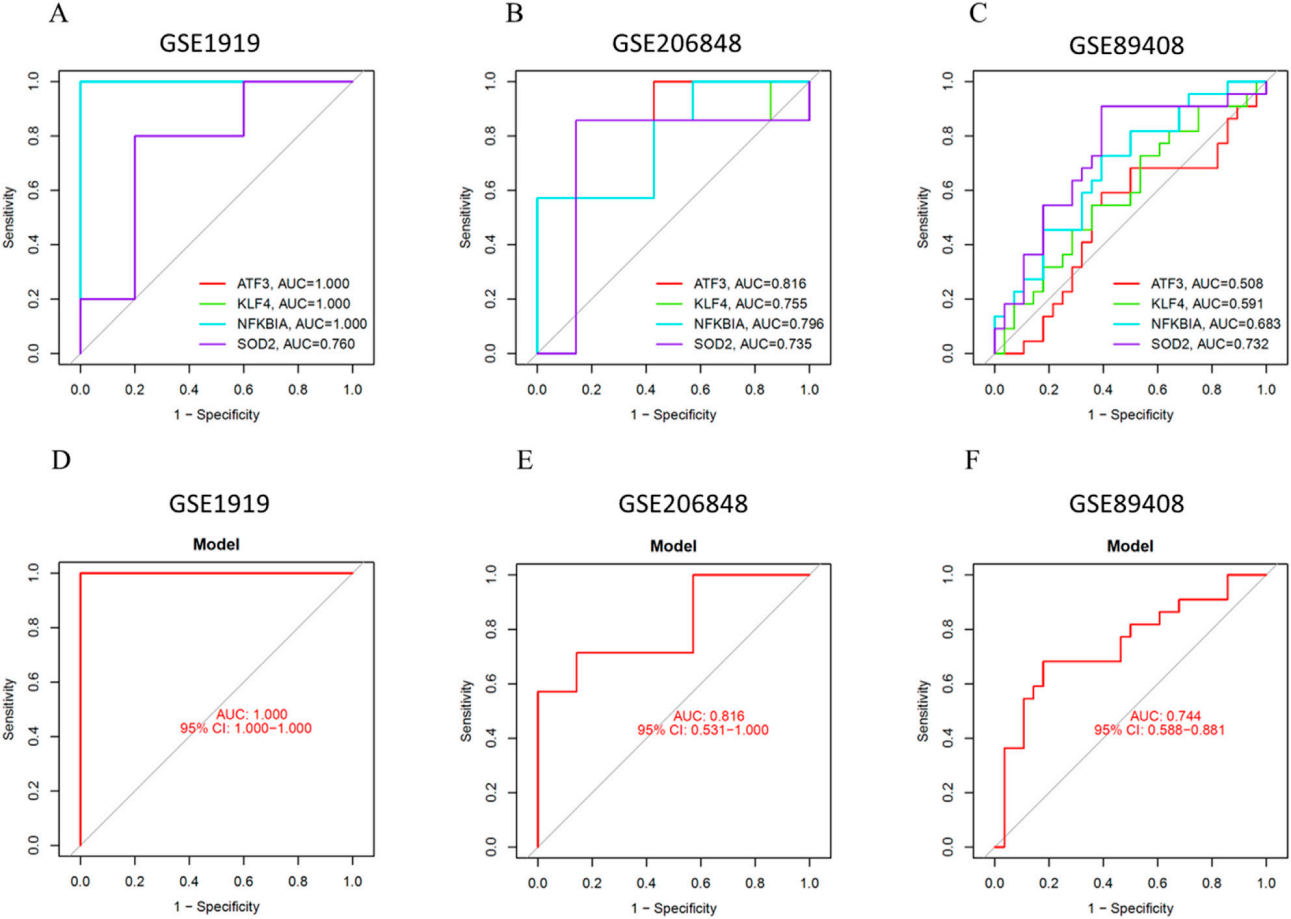
Validation of the Prediction Model. **(A)** ROC curve of key genes in GSE1919. **(B)** ROC curve of key genes in GSE206848. **(C)** ROC curve of key genes in GSE89408. **(D)** ROC curve of the prediction model in GSE1919. **(E)** ROC curve of the prediction model in GSE206848. **(F)** ROC curve of the prediction model in GSE89408.

To further assess the accuracy of the prediction model, we validated the diagnostic performance of the four key genes using three OA synovial tissue datasets. First, in the GSE1919 dataset, the AUC values for ATF3, KLF4, NFKBIA, and SOD2 were 1.000, 1.000, 1.000, and 0.760, respectively (Figure 8A). The AUC value of the prediction model was 1.000 (95% CI: 1.000–1.000) (Figure 8D), demonstrating a high diagnostic efficacy. Next, in the GSE206848 dataset, the AUC values for ATF3, KLF4, NFKBIA, and SOD2 were 0.816, 0.755, 0.796, and 0.735, respectively (Figure 8B). The AUC value of the prediction model was 0.816 (95% CI: 0.531–1.000) (Figure 8E). Furthermore, in the GSE89408 dataset, the AUC values for ATF3, KLF4, NFKBIA, and SOD2 were 0.508, 0.591, 0.683, and 0.733, respectively (Figure 8C). The AUC value of the prediction model was 0.744 (95% CI: 0.585–0.881) (Figure 8F).

### *In vitro* qRT-PCR experimental validation

**FIGURE 9:**
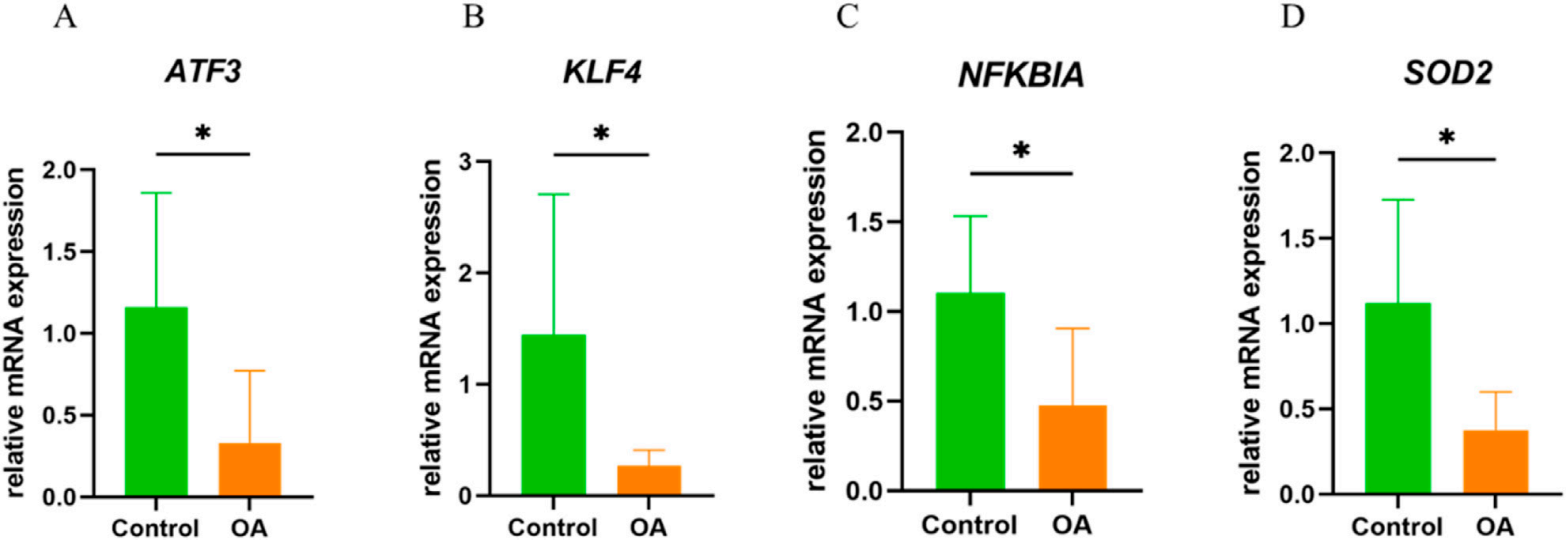
*In Vitro* qRT-PCR Experimental Validation. **(A)** mRNA expression levels of ATF3 in normal and OA synovial tissue. **(B)** mRNA expression levels of KLF4 in normal and OA synovial tissue. **(C)** mRNA expression levels of NFKBIA in normal and OA synovial tissue. **(D)** mRNA expression levels of SOD2 in normal and OA synovial tissue. *P < 0.05.

The qRT-PCR results showed that, compared to normal synovial tissue, the expression levels of ATF3, KLF4, NFKBIA, and SOD2 were significantly downregulated in OA, which is consistent with the results from the aforementioned bioinformatics analysis (Figures 9A–D).

### Immune cell infiltration analysis

**FIGURE 10:**
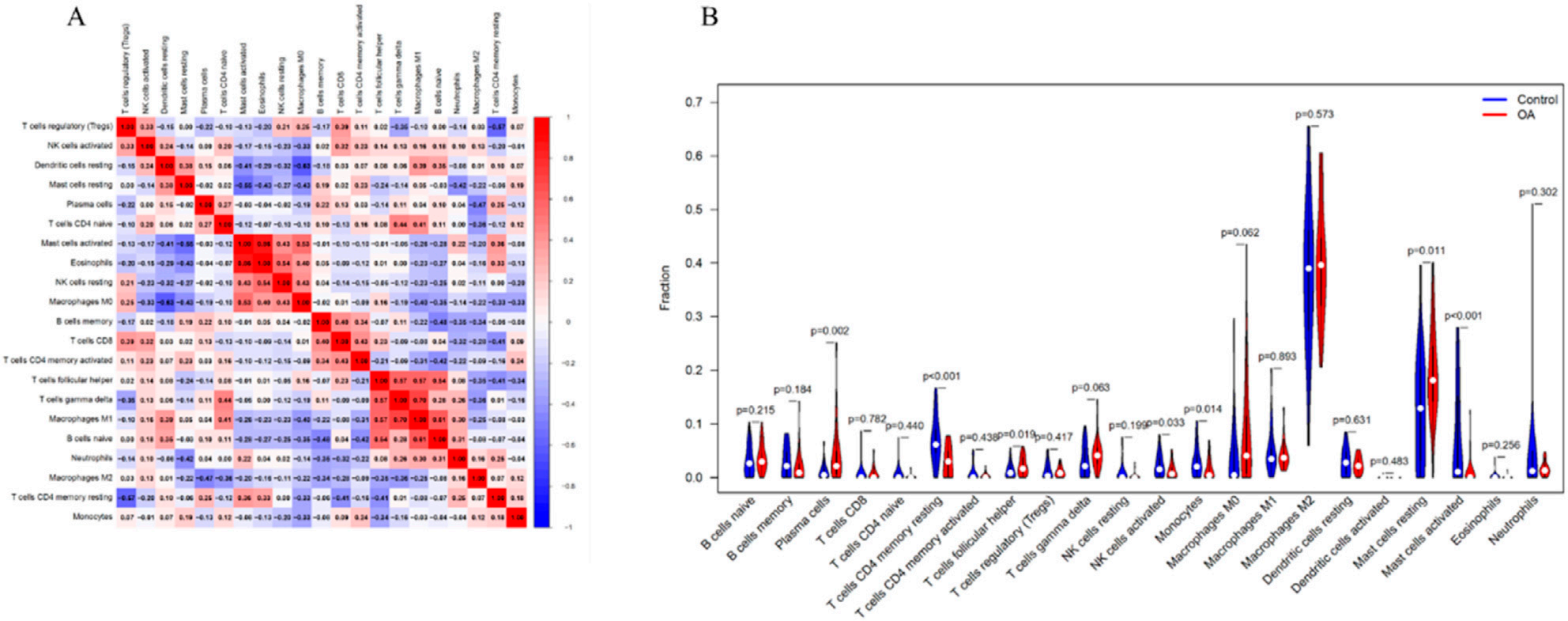
Immune Cell Infiltration Analysis. **(A)** Heatmap of correlations between immune cells. **(B)** Differential levels of immune cells between the normal control group and OA group. P < 0.05 was considered statistically significant.

Based on the CIBERSORT algorithm, the correlation between immune cells revealed that Macrophages M0 were negatively correlated with resting Dendritic cells, while Macrophages M1 were positively correlated with gamma delta T cells (Figure 10A). Additionally, immune cell differential analysis between the OA group and normal controls showed that Plasma cells, follicular helper T cells, and resting Mast cells were significantly elevated in OA. In contrast, memory resting CD4^+^ T cells, activated NK cells, Monocytes, and activated Mast cells were significantly decreased in OA (Figure 10B).

### Correlation analysis between key genes and immune cells

**FIGURE 11:**
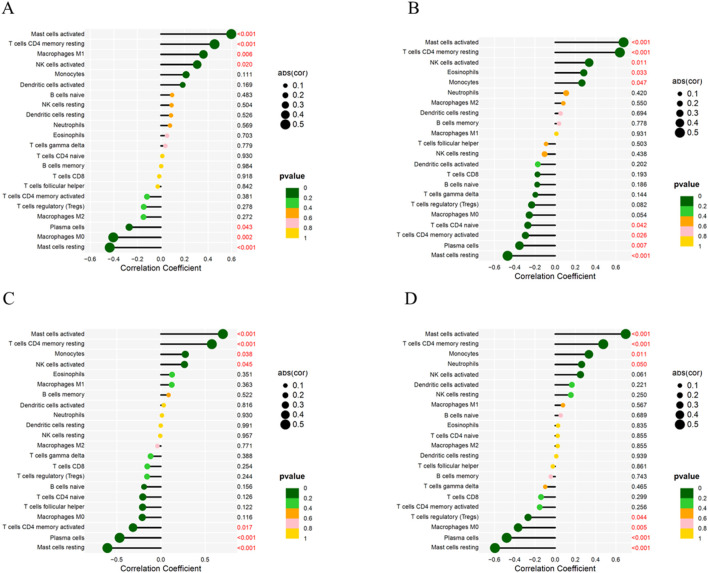
Correlation Between Key Genes and Immune Cells. **(A)** Correlation between ATF3 and immune cells. **(B)** Correlation between KLF4 and immune cells. **(C)** Correlation between NFKBIA and immune cells. **(D)** Correlation between SOD2 and immune cells.

The correlation analysis results showed that ATF3 was positively correlated with activated Mast cells, CD4 memory resting T cells, M1 Macrophages, and activated NK cells, and negatively correlated with Plasma cells, M0 Macrophages, and resting Mast cells (Figure 11A). KLF4 was positively correlated with activated Mast cells, CD4 memory resting T cells, activated NK cells, and Eosinophils, and negatively correlated with naïve CD4 T cells, activated memory CD4 T cells, Plasma cells, and resting Mast cells (Figure 11B). NFKBIA was positively correlated with activated Mast cells, CD4 memory resting T cells, Monocytes, and activated NK cells, and negatively correlated with activated memory CD4 T cells, Plasma cells, and resting Mast cells (Figure 11C). SOD2 was positively correlated with activated Mast cells, CD4 memory resting T cells, Monocytes, and Neutrophils, and negatively correlated with regulatory T cells (Tregs), M0 Macrophages, Plasma cells, and resting Mast cells (Figure 11D).

## Discussion

Osteoarthritis (OA) is a degenerative joint disease closely associated with aging (Liao et al., 2024; Li et al., 2024). Cellular senescence is a key hallmark of the aging process and is particularly evident in OA, where features of cellular senescence can be observed (Loeser et al., 2016). Although growing evidence suggests a close association between aging and OA, the underlying pathogenic mechanisms remain unclear (Yan et al., 2023). Therefore, this study aims to explore the role of aging-related genes in OA using bioinformatics and machine learning algorithms, providing new insights into the pathogenesis and potential treatment strategies linking aging and OA.

In this study, 34 DEARGs were identified from synovial tissue samples of normal controls and OA patients using bioinformatics analysis. Subsequently, four key genes (ATF3, KLF4, NFKBIA and SOD2) were identified through PPI network analysis, LASSO regression, and random forest algorithms. An OA prediction model constructed based on these genes demonstrated good diagnostic performance. Furthermore, qRT-PCR experiments confirmed that all four genes were significantly downregulated in OA synovial tissues, consistent with the computational analysis. These findings may offer new insights into therapeutic strategies for elderly patients with osteoarthritis.

Activating Transcription Factor 3 (ATF3) is a stress-induced transcription factor that plays a crucial role in the cellular response to various stresses, regulating the interaction between cell metabolism, immunity, and inflammation to maintain cellular homeostasis (Liu et al., 2024; Li et al., 2023). Interestingly, ATF3 can regulate the expression of matrix metalloproteinase 13 (MMP13), leading to the degradation of type II collagen, loss of extracellular matrix, and disruption of cartilage homeostasis, thereby potentially contributing to the aging process in OA (Chan et al., 2017). Li et al. found that the PR11-364P22.2/ATF3 regulatory axis can modulate the catabolic activities of cartilage tissue and chondrocytes induced by IL-1β, thereby influencing the progression of OA (Li et al., 2021). Moreover, ATF3 deficiency can suppress the expression of inflammatory cytokines in OA chondrocytes, thereby playing a role in the onset and progression of OA (Iezaki et al., 2016). In this study, we also observed a significant decrease in ATF3 levels in OA synovial tissue, providing new insights into the role of ATF3 in OA.

Kruppel-like factor 4 (KLF4) is a transcription factor characterized by a zinc-finger (ZNF) structure, and it is implicated in the pathogenesis of various inflammatory diseases, including inflammatory bowel disease, OA, kidney inflammation, pneumonia, and neuroinflammation (Liang et al., 2024). In addition, KLF4 has been confirmed to be involved in the aging process of OA. Studies have shown that KLF4 upregulates the expression of MMP13 in chondrocytes, potentially providing a new therapeutic target for OA (Takeuchi et al., 2020). In OA, KLF4 can regulate the catabolic and inflammatory responses of chondrocytes and synovial cells, thereby preventing joint tissue destruction and inflammation (Kawata et al., 2022). Additionally, the study found that KLF4 expression is decreased in OA synovial tissues, which may offer a theoretical basis for exploring the pathogenesis of OA-related aging.

NFKBIA is a member of the NF-κB inhibitor family, and it plays a crucial role in regulating NF-κB activity and modulating inflammatory responses (Tang et al., 2018; Wang et al., 2020). In addition, NFKBIA may be associated with the senescence of chondrocytes and synovial cells, making it a potential therapeutic target for OA (Chien et al., 2011). In OA synovial fibroblasts, suppression of NFKBIA overexpression has been shown to reduce inflammation by decreasing the levels of MMP13 and ADAMTS4 (Bondeson et al., 2007). Lin et al. found that modulating the NF-κB signaling pathway can downregulate MMP13 expression in synovial fibroblasts, suggesting a potential approach for treating inflammatory arthritis (Lin et al., 2011). Interestingly, Xu et al. reported that inhibiting the activation of the NF-κB pathway exerts anti-inflammatory effects on chondrocytes, thereby alleviating OA (Xu et al., 2021). Although NFKBIA plays a crucial role in OA-related aging, its underlying mechanisms remain unclear and require further investigation.

Superoxide dismutase 2 (SOD2) is a mitochondrial antioxidant enzyme that catalyzes the conversion of superoxide radicals into oxygen and hydrogen peroxide, thereby protecting cells from oxidative damage (Zhou et al., 2023). Increasing evidence suggests that SOD2 plays a significant role in cellular senescence associated with OA (Bolduc et al., 2019). Jian et al. found that modulating the SIRT3/SOD2 pathway can inhibit chondrocyte senescence, potentially offering a new therapeutic avenue for OA (Jiang et al., 2025). Interestingly, downregulation of SOD2 leads to oxidative stress and mitochondrial dysfunction in chondrocytes, which may be a contributing factor to OA pathogenesis (Gavriilidis et al., 2013). Furthermore, this study found that SOD2 is significantly downregulated in synovial tissue, providing new insights into the connection between OA and aging.

There are several limitations in this study. First, although this study validated gene expression using clinical synovial tissue samples, the relatively small sample size may affect the reliability of the results. In addition, clinical confounding factors such as age, medication use, surgical history, and lifestyle may have influenced gene expression. Therefore, future studies should include a larger number of samples with clearly defined clinical characteristics to verify and extend the current findings. Second, the validation experiments conducted were relatively limited. Future studies should include animal models and *in vitro* cell experiments to further verify the roles of these genes. Moreover, synovial tissue exhibits cellular heterogeneity, and this study did not clarify whether these genes are expressed in specific cell types. This limitation could be addressed through single-cell RNA sequencing or immunohistochemical analysis in future research.

## Conclusion

In this study, we employed bioinformatics and machine learning to identify four key aging-related genes associated with OA and constructed a predictive model for OA. These genes may play important roles in the pathogenesis of OA-related aging and offer new directions for future prevention and treatment strategies.

## Data Availability

The original contributions presented in the study are included in the article/Supplementary Material, further inquiries can be directed to the corresponding author.
